# Strategies to improve the quality of midwifery care and developing midwife-centered care in Iran: analyzing the attitudes of midwifery experts

**DOI:** 10.1186/s12884-022-04379-7

**Published:** 2022-01-16

**Authors:** Shahla Khosravi, Farah Babaey, Parvin Abedi, Zohreh Mazaheri Kalahroodi, Saeideh Sadat Hajimirzaie

**Affiliations:** 1 Department of Community Medicine, Faculty Member of Medicine School, Tehran University of Medical Sciences, Tehran, Iran; 2 Health Policy, Ministry of Health and Medical Education, Tehran, Iran; 3 Midwifery Department, Menopause Andropause Research Center, Ahvaz Jundishapur University of Medical Sciences, Ahvaz, Iran; 4 Health System Reform, Iran University of Medical Sciences, Tehran, Iran; 5 School of Nursing and Midwifery, Shahroud University of Medical Sciences, Shahroud, Iran

**Keywords:** Midwifery care, Qualitative study, Women’s health, Quality

## Abstract

**Background:**

In recent years, extensive studies have been designed and performed in the context of providing midwifery care in developed countries, which has been unfortunately neglected in some low resources and upper middle-income countries such as Iran. This study was conducted to identify the best strategies for improving the quality of midwifery care and developing midwife-centered care in Iran.

**Methods:**

This was a qualitative study using focus group discussion and content analysis method. Data were collected from 121 participants including midwifery board members, gynecologists, heads of midwifery departments, midwifery students, in charge midwives in hospitals, and midwives in the private sector. Focused-group discussions were used for data collection, and data were analyzed using content analysis method.

**Results:**

The main themes extracted from the participants’ statements regarding improving the quality of midwifery care were as follows: Promotion and development of education, Manpower management, Rules, and regulations and standards for midwifery services, and Policy making.

**Conclusion:**

This study showed that to improve midwifery care, health policy makers should take into account both the quality and quantity of midwifery education, and promote midwifery human resources through employment. Furthermore, insurance support, encouragement, supporting and motivating midwives, enhancing and improving the facilities, providing hospitals and maternity wards with cutting-edge equipment, promoting and reinforcing the position of midwives in the family doctor program, and using a referral system were the strategies proposed by participants for improving midwifery care. Finally, establishing an efficient and powerful monitoring system to control the practice of gynecologists and midwives, promoting the collaborative practice of midwives and gynecologists, and encouraging team-work with respect to midwifery care were other strategies to improve the midwifery services in Iran. Authorities and policymakers may set the stage for developing high quality and affordable midwifery care by relying on the strategies presented in this study.

**Supplementary Information:**

The online version contains supplementary material available at 10.1186/s12884-022-04379-7.

## Background

Despite its direct effect on community development health of the mother and baby has not received due priority in every society, and this accounts largely for the reason why maternal mortality remains an interactable problem in many countries [1]. The importance of maternal and child health is so paramount that the World Health Organization (WHO) has particularly emphasized the development of the health of mother and baby in its 2020 report [2]. Among the various groups involved in the health of the mother and baby, midwives have the highest chance to provide the best care for promoting maternal and their health [3]. In midwifery care services, a large number of women are continuously in touch with midwives, which makes early detection of mothers with certain needs easier [4].

Every day, around 810 women die from pregnancy-related causes, most of which are preventable and happen in low and middle-income countries [5]. In addition, many women who survive suffer from the negative consequences of pregnancy and childbearing and may experience long-term disabilities [6]. In developed countries, on the other hand, due to the high-quality and free midwifery care, the shining role of midwives in maternity care and the better supervision of referral system have resulted in lower maternal mortality rate compared with developing and low-resources countries [6, 7].

The results of previous studies confirm that babies who are born to mothers without antenatal care are almost more likely to die and be exposed to childbearing consequences compared to those born to mothers with good antenatal and midwifery care [8]. According to the WHO report, in areas where more than 75% of births are performed by midwives, there is a lower rate of maternal mortality, and this rate has even decreased by 75% in areas where employed educated midwives provide care [9].

According to a study on childbirth centers in Australia, antenatal death rate in these centers was lower compared with hospital units for healthy pregnant women [10]. Another study showed that women who received woman-centered care from midwives in their pregnancies and deliveries had pleasant and good experiences in childbearing with lower complications compared to medical-oriented care services [11]. In Germany, a study showed a lower rate of epidural anesthesia and episiotomy as well as reduced length of the second stage of labor in women who received midwifery-led care in comparison to those under obstetrician care [12].

Notwithstanding, the results of many studies in Iran show that midwifery care is not given as much value as it is in other countries. Despite the high level of Iranian midwifery education, women do not receive proper midwifery care in this country. One of the underlying causes for this is lack of insurance coverage for midwifery care, making women opt for a gynecologist’s service that is under full insurance coverage. Gynecologists, more often than not, tend to impose some unnecessary interventions in antenatal care and during childbearing, which may increase the rate of caesarean section [13, 14]. Despite the reduction of maternal and neonatal mortality rate in recent years in Iran (16 maternal deaths per 100,000 live birth and 8.6 neonates deaths per 1000 live births respectively) [15], the rate of cesarean section in this country has increased (48% in general, and up to 87% in some private hospitals) [16]. As gynecologists are in-charge of delivery rooms in Iran, and midwives should work under the supervision of gynecologists or residents, it is not possible for midwives to work and make independent decisions, midwives are not able to make decisions regarding physiological delivery and reduction of interventions.

One way that can reduce maternal mortality and disability is to prevent unnecessary intervention during childbirth through improving the pre-birth service centers and providing high quality and accessible services for pregnant women [17]. Unlike teaching hospitals, in public health centers, midwives are responsible for providing prenatal care to pregnant women, but they do not have the right to refer women to a gynecologist if a problem is diagnosed, and this is done instead by a general practitioner.

Given the fact that midwives play a significantly critical role in providing primary care for saving mothers’ life, it is necessary to improve the quality of midwifery care. In recent years, extensive studies have been designed and performed in developed countries in the context of providing midwifery care, which has been unfortunately neglected in some low-resources and upper middle-income countries such as Iran [18]. Hence, the purpose of the current study was to identify the main strategies for improving the quality of midwifery services and developing midwife-centered care in Iran.

## Methods

This was a qualitative study using focus group discussion approach. The reason for adopting this approach was assembling individuals (midwives in different position of policy making) in a group where they were able to exchange their ideas with the aim of drawing conclusions from personal experiences and beliefs [19].

The purposive sampling method was used for selecting the participants. In this method, participants are chosen from among those who have the most and richest information. Participants of the present study included 121 individuals from various positions. The characteristics of participants are presented in Table 1. Participants were selected due to their high experience and sufficient knowledge in their work and midwifery services in Iran.

**Table 1:** Characteristics of participants in the study

Kind of professional activity	Number of focus group discussion	Number in each group	Education	Age range (y)	Average years in the profession
Midwifery board member	1	11	Master degree and PhD	34–58	19
Gynecologist	1	5	Clinical specialist	38–57	20
Head of midwifery department	5	6	Master degree and PhD	30–52	16
In charge midwives in the educational hospitals	3	5	Master degree and PhD	32–50	18
In charge midwives in the non-educational hospitals	3	5	Master degree and PhD	29–46	18
Head of midwifery department in non-governmental universities	1	5	Master degree and PhD	28–47	14
Midwives in medical system with work licenses	4	5	Master degree and PhD	30–45	13
Midwives in the headquarters of the ministry of health	1	5	Master and PhD	30–48	17
Midwifery students	3	5	The third and fourth year of education	20–26	3

The inclusion criteria included familiarity with midwifery services, having at least one year of work experience in midwifery and gynecology settings or two years of education in the midwifery field, willingness to participate in focus group discussions, and the ability to communicate and provide comments.

Sampling continued until data saturation. In the current study, saturation was obtained with 110 participants. However, to ensure complete saturation, the researchers continued participant selection up to 121 individuals. Moreover, attempt was made to select a variety of participants in terms of age, working place, employment status, work experience, educational attainment, and occupational position to increase variety of data.

The setting of this study was the midwifery office of the Health Minister Advisor in Midwifery Affairs and Akbarabadi Hospital in Tehran. The main reason for choosing these places was simple access of all participants to these places. Akbarabadi Hospital is a public hospital located in south of Tehran (capital of Iran).

Focus group discussions were used for data collection. Two authors (SK and FB) who held PhDs in Reproductive Health and Health Policy, respectively conducted all focus group discussions. In each session, one acted as a facilitator, and the other was responsible for taking notes and audio recording [20]. Both researchers had previous experiences in conducting focus group discussions, were interested in the topic under study, and they aimed to resolve the obstacles associated with using midwifery-led care in Iran. One of the interviewers (SK) was a faculty member in Tehran University of Medical Sciences and the other (FB) was an employee in Iran Ministry of Health. All interviewers and participants were female.

All participants were invited by prior arrangements and formal invitation letters. Except for the focus group discussion with midwifery board members that included 11 participants, each focus group discussion consisted of 5–6 people and lasted from 60 to 90 min. For Head of midwifery department, in charge midwives in the educational hospitals, in charge midwives in the non-educational hospitals, midwives in medical system with work licenses, and midwifery students groups more than one focus group discussion was performed.

At the beginning of the discussion, the interviewers were introduced to group members, and the goals of the focus group discussion were explained. The discussion started by asking about the status of midwifery at setting such as hospitals or training centers. Some of common questions asked in the focus groups were as follows: How do you describe the status of midwifery service in Iran? What are the barriers to providing high quality midwifery services? What are the solutions for improving midwifery services and their quality?

In total, we had 22 focus groups. The focus group discussion guide was developed for this study is presented in Additional File 1. The participants’ statements were recorded upon their consent using a voice recorder, while one of the interviewers was also taking notes during group discussions. After conducting 22 focus group discussions, the researchers reached data saturation (i.e. no more new ideas were provided by participants). All interviews were carefully listened to and transcribed.

To analyze the data, we used content analysis, a method of identifying, analyzing, and reporting patterns (themes) existing within the text which is very pertinent in analyzing qualitative data [21]. Data coding was done by two of the researchers. The analyses and coding stages were as follows:

Familiarity with the text (reading the texts several times - data immersion), identification and extraction of primary codes (identification and extraction of data related to the original codes), identification of themes (inserting the extracted source code in related themes), reviewing and completing the identified themes, naming and defining the themes, and ensuring the reliability of the extracted codes and themes (reaching an agreement between the two coders through discussion). Peer check and immersed data were used for data consistency. The initial codes derived from focus group discussions were checked by some of participants, who made some corrections.

To comply with the ethical considerations in this study, we obtained informed consent from all participants, and they also had the right to withdraw from the study whenever they wanted. In addition, the research objectives were explained to the participants at the outset of the study (Additional File 2).

## Results

In this study, 121 professionals participated all of whom had the experience of working in different fields of midwifery practice and were involved with the research topic. The details of the participants are shown in Table 1.

According to the results, 259 primary codes were derived from the focus groups. After elimination of similar codes, this number decreased to 162. In the next stage, similar codes were integrated with each other yielding 66 codes that were grouped into four themes as follows: Promotion and development of education, Manpower management, Rules, regulations, and standards for midwifery services, and Policy making, that each theme had three subthemes (Table 2). In the following the themes subthemes with some examples are enumerated:

**Table 2:** Strategies for promoting midwifery care in Iran from the perspective of midwifery experts (*N* = 121)

Area		
Promotion and development of education	University	Paying special attention to the quality of midwifery student’ education and selecting their instructors based on specific criteria. The education of midwifery students should be revised so that they are committed and efficient after graduation. Increasing the training course of midwifery students to 5.5 years Use of midwives with theoretical and clinical experience and for education of midwifery students.
	Graduate and in-service training	Holding a training course on childbirth preparation for midwives in the health centers. Training of new employed staff Continuous and codified training for midwives in the maternity ward Periodic training for midwives of health centers for proper control of mothers of prenatal education.
	Educating the community	Promote natural childbirth using the media and install advertising banners Educating pregnant mothers in health centers about natural childbirth Obligation to teach the benefits of natural childbirth in pre marriage counseling classes Creating a culture about natural childbirth and the complications of cesarean section, especially on television
Manpower management	Management of midwifery forces	➢Creating new positions of employment for midwives ➢Employment based on skills, efficiency and scientific knowledge ➢Using midwives in clinical fields ➢Providing permission to employ midwives for clinical work
	Midwifery management	➢Stablishing of midwifery supervisor ➢Presence of midwives in managerial positions ➢Attending to maternal mortality committess
	Supportive and incentive systems	➢Payments of natural births to midwives in hospitals ➢Legal protection of midwives in the private sector ➢Reform of payment system ➢Increase the difficulty of midwifery job to 100% ➢Creating motivation by assigning of in charge midwife to the head of delivery ward
**Rules and standards for midwifery services**	Regulations on the professional qualifications of midwives	➢Revise the midwifery duties ➢Full implementation of the letter description section of midwife duties
	Audit and supervision	➢Existence of reprimand tools for specialists who perform elective cesarean section or make unscientific decisions ➢Establish a strong system for monitoring cesarean section indications ➢Supervise the implementation of letters related to midwifery and protocols
	Equipment and Facilities	➢Maternity facilities should be rehabilitated ➢Equipping hospitals with 24-h physiological delivery wards ➢Standardization of delivery ward space in all hospitals ➢Modification of physical environment of hospitals for rapid access of clients in time of emergencies
**Policy making**	Organization	➢Determining the appropriate position of midwives in the family physician and referral program ➢Hospitals should not be self-government
	Compliance with the general policies	➢Using the words of the Supreme Leader of Iran in relation to population growth and natural childbirth to decrease cesarean section ➢Improving lifestyles and changing beliefs in order to implement general health policies
	Interdisciplinary activities	➢Development of team activity protocols for midwives and gynecologists ➢Increase interaction between private universities and the Ministry of Health ➢Holding periodic symposiums between midwives of the private and national universities.

### Promotion and development of education

According to the participants’ views in different groups, the main concern of the participants was to improve the quality of midwifery care. The participants also stressed developing and improving the educational status of midwifery students and graduate midwives in order to increase their capabilities and promote the knowledge of the recipients of service about their reproductive rights. According to one of the participants, “Midwifery training programs should be regularly reviewed, especially as far as training is concerned, so that after graduation the trainees can later provide independent, committed and efficient midwifery services.”

#### Academic education

One of the midwifery board members and a gynecologist emphasized the need to empower faculty members in clinical education as a way to improve the quality of midwifery services. She maintained: “midwifery faculty members should be selected according to specific and standard criteria, and even if they need, they should pass mandatory vocational courses at the beginning of their enrollment, as well as in-service training courses”. One of the private sector midwives stated: “It seems that in order to increase the quality of midwives’ work, graduates of this field should work with a temporary degree for a while, and after ensuring the quality of their work, they may receive the main degree”. Another participant added: “Midwifery courses should be regularly assessed, especially clinical courses, so that the students can provide independent, committed, and efficient midwifery services after graduation”.

#### Graduate and in-service training

Although improving the quality of midwifery education for students is very important, in-service training of midwives should not be neglected. One of the participants (a gynecologist) from the private sector stated: “In order to improve midwifery services, it is possible to make working with a midwifery license conditional upon passing some clinical courses, or to accredit midwifery license on the condition of passing some courses in maternity wards. The training services should be offered with the help of a scientific association or other skill training centers.” This participant even emphasized that “In order to coordinate the health sector with the hospital care or for the proper control of pregnant women, passing some of these courses is necessary, even for midwives working in the public health centers”.

#### Educating the community

Several groups of participants stated that one of the ways to promote midwifery services is to educate both service recipients and the community to be aware of the real needs and expectations and fertility rights, and to enhance their understanding of midwifery care and the options that they have. The participants stressed that service recipients should make free and informed decisions by receiving comprehensive information and that childbirth should become a pleasant experience for them.

One of the participants in the midwifery service group stated: “We should have a consistent program to be implemented that through mass media in order to promote natural childbirth or teach safe pregnancy issues in premarital education classes. By educating women and enhancing their decision-making skills, and perhaps even educating their husbands and families, we can improve the quality of the midwifery services they need”.

### Manpower management

The following subthemes were emerged:

#### Supplying of skilled midwives

One of the important issues in manpower management is to supply the necessary midwifery staff to provide midwifery services. One of the participants proposed the following solution: “The number of midwives in hospitals is not proportional to the workload of midwives because either there are few vacancies for midwives in hospitals (the positions are dedicated to hire other staff) or the officials cannot allocate financial resources for more midwifery staff; therefore, they use fewer midwives to do a greater amount of work. For this reason, we are constantly facing a shortage of staff in the midwifery sector, and it is necessary to create new midwifery job titles to provide quality services”.

#### Midwifery management

Participants in this study pointed out a number of strategies by which midwives can enhance their practice to improve the health of mothers and neonates. One of the in-charge midwives stated that having a midwifery supervisor is one way to improve the quality of midwifery services in hospitals. She said, “in hospital, each profession has a supervisor, so midwives need a supervisor too. Because we do not have a supervisor, when it comes to calculating salaries and benefits, midwives are at a disadvantage. When the authorities estimate the manpower, midwives are not estimated accurately in proportion to their workload. When something wrong happens, there is no professional manager who can handle it”.

Several participants in the group of in-charge midwives from university and non-university hospitals also raised this issue regarding the position of midwives. “Midwives in the delivery room and postpartum ward have the greatest role in reducing maternal and neonatal mortality rates, even the rate of infection of the baby and morbidity of mothers. Midwives should be a key-members in maternal mortality committees; they can comment on process reform”.

Another of the proposed solutions was the presence of an experienced midwife in the quality improvement office to do accreditation of midwifery practice.

#### Support and incentive systems

One of the key points in management of midwifery manpower is specifying incentives and paying attention to the factors affecting burnout due to special working conditions in the delivery room and care of mothers and infants, as well as supporting human resources and promoting natural childbirth.

Participants who worked in maternity wards also proposed a number of solutions for this issue. One of them stated: “The midwife not only performs the delivery but also gives all the care needed during the labor. When officials want to pay incentives for natural childbirth, the payment to the midwife is very low, especially in non-university hospitals. Authorities should propose methods to motivate midwives. Not only should the payment system for midwives be modified, but a fair payment plan should also be implemented consistently in all centers”.

One of the issues raised by many participants which can be effective as a supportive strategy was taking into account the work difficulty of midwives. One of the participants stated: “everyone knows that the delivery room has special conditions given the noise pollution, constant contact with blood and discharge during childbirth, and the stress caused by the emergency condition of the mother and the baby. Therefore, the delivery room should be considered as an emergency department, and emergency fees should be paid to midwives in delivery rooms.”

### Rules and standards of midwifery services

Good management based on the participation of all service providers and managers leads to improved service quality. Quality is one of the most important criteria for satisfying mothers and the main mission of the health system is to provide quality care and meet their needs and expectations.

Improving the quality of midwifery services can reduce overall treatment costs, provide effective teamwork, meet the expectations and needs of women and families, and improve indicators related to maternal and infant health [22].

### Regulations on professional competency of midwives

To provide better and more effective midwifery services, the duties of midwives and the boundaries of their services should be clearly defined at different times and in different positions. One of the participants, who had a long work experience, said, “The competence of the midwifery has expanded, but it should also be reviewed in light of the vast scientific advances in this regulation, and of course, relevant managers and policymakers should be aware to make decisions and develop clinical guidelines.”

Setting regulations on the competence needed for different midwifery qualifications brings about coherent of services. One of the effective solutions proposed by the participants was to compile these regulations with specific tasks for each level and for different positions, even for the faculty members.

One of the midwifery managers that had 15 years of service said:

“In a teaching hospital, the job description of the midwifery instructor should be specified and there should be a system for monitoring the performance of the instructor. The qualifications for the instructors should be clear. They should be familiar with the relevant regulations.”

### Audit and supervision

According to one of the faculty members, “Not only should the duties of midwives be defined, but also specific rules for working in hospitals should be set; for example, what skills midwives should have, and what in-service training they should receive”.

Another faculty member said, “A faculty member should continuously refresh their clinical skills. For example, a clinical course on normal vaginal delivery without interventions should be offered to all those who work in the delivery room, even the instructors.”

Setting standards for maternal and neonatal care in delivery and postpartum wards was also one of the solutions proposed by the participants.

One of the participants from a university hospital stated that, “One of the solutions is setting service standards and clinical guidelines. There should be a standard for the services provided to determine the effectiveness of the service. Of course, there are standards for each department. This is because the provision of midwifery services in a university hospital with the presence of residents is different from a non-university hospital.”

One of the participants in the group of midwifery educators stated: “Students should be familiar with the standards of care because they are going to work here later. Instructions that are issued for midwifery services must be taught to midwifery students and residents. Important features of a clinical education should include all the effective learning conditions, and there should be no gap between theoretical and clinical courses.”

According to the midwives working in the wards in our study, one of the ways to improve the quality of services is for everyone who provides care in the midwifery service to be familiar with the standards. Of course, revision of the standards, especially the registration of information, is also necessary. One of the participants said: “The system of registration of births should be reformed. The data should be documented and evaluated by the General Directorate of Midwifery and sent to midwives and specialists. The deputy director of treatment should review the statistics, instructions, etc. to improve services.”

### Equipment and facilities

One of the ways to improve the quality of services is the providing appropriate physical conditions and equipping delivery wards according to standards.

A participant with a long work experience stated: “When we started working in the delivery room, we could provide good services even though the physical space was not equipped as it is now, because we had a small number of clients”. However, with the advancement of science and the promotion of skills and expectations of women, these conditions are not enough to provide standard care, and all rooms must be equipped to serve a one-by-one service by a midwife”.

One of the important issues that proposed by another participant was: “ Educational opportunities to improve midwifery education and creativity should be provided. In this regard, by identifying educational spaces and making them flexible compatible with student interactions, the possibility of creativity and growth over time will increase that this play a significant role in improving the quality of education.”

### Policy making

#### Organization

One of the solutions that was proposed in focus group discussions was that the opportunity of increasing the quality of services should be provided for the service provider at both the hospital setting and the health centers, and the position of the midwife in the referral system should be defined. One of the midwives said: “In order to achieve midwifery services, the position of midwives in the referral system should be clear, and midwives should be defined in the referral processes of insurance organizations. If we are not visible in the referral system, how can midwifery services be taken into account? Of course, the support of senior managers is also important.” Another in-charge midwife at the Ministry of Health stated, “Due to the efforts of the Ministry of Health in establishing a family doctor system, midwives should be at the referral level, in this case it will be possible to sign a contract with private sector midwives.”

#### Compliance with general policies

One of the main policies of Iran in recent years has been population policies focusing on increasing fertility rate, improving life expectancy, providing healthy nutrition, and preventing social harms. Based on these policies, a variety of roles are assigned to various institutions, organizations and groups that provide services and care to the society. Midwives can play an effective role in implementing these policies by providing services for healthy fertility, providing maternity care, promoting natural and physiological childbirth, and empowering women to strengthen the family. In our group discussions, participants explained the appropriate policies and programs for midwifery services. “Declaration of policies to encourage childbearing should be properly planned and this opportunity should be seized to strengthen midwifery services,” said a midwife at the Ministry of Health.

#### Interdisciplinary activities

Establishing inter-sectoral relations can be a very effective help in the development of services. This includes the development of packages on team delivery services for gynecologists and midwives, cooperation between public and private education systems (e.g. midwifery education by the Islamic Azad University), and establishment of a link between midwifery services in the treatment system and health network.

## Discussion

This study aimed to identify strategies for improving the quality of midwifery care and developing midwife-centered care in Iran. According to our results, four themes were extracted from the participants’ statements including Promotion and development of education, Manpower management, Rules, regulations, and standards for midwifery services, and Policy making.

The Promotion and development of education included the following subthemes: Academic education, graduate and in-service training, and educating the community. In our study, enhancement of the quality of midwifery education was among the most important issues emphasized by the vast majority of the participants. During recent years, some of the studies conducted in Iran have admitted that midwifery education is faced with many problems [20]. One of the reasons contributing to these problems is that the midwifery student is not allowed to gain experience in university hospitals because of the presence of residents. The Bachelor of Midwifery training program is a four-year program that provides approximately 1200 h of clinical work; in addition, a midwifery student has to perform 60 deliveries independently in order to graduate.

In teaching hospitals, midwifery instructors are required to train a midwifery student under the supervision of a resident or a gynecologist, and this may prevent the midwifery student from learning physiological delivery well. However, training in non-university hospitals does not have these challenges.

The quality of midwifery education has a profound influence on the presentation of clinical midwifery skills. Newly graduated midwives should have already obtained the minimum clinical and professional skills so that they can perform midwifery duties, improve the care of mothers and babies, and ultimately enhance the health of the community [23]. Also, educational curricula must be evaluated at certain junctures and based on acceptable standards.

[24].

Training after graduation and in-service training was the second subtheme of the first theme, that was emphasized by participants in the focus groups. Gavine et al. conducted a systematic review on pre-service and in-service education of maternal and newborn care providers in low and middle-income countries. Their results showed that although there is some evidence that focuses on training on emergencies during labor and delivery, there is lack of sufficient programs for education of midwives according to international standards with the full scope of competencies [25]. The results of the present study are in line with those of Gavine et al.

Educating the community was the third subtheme of the first theme in this study, and it emphasizes training midwives and empowering them so that they can meet the needs of women and children in the community. One of the necessities and fundamental strategies for effective midwifery services is empowering midwives to be capable of communicating with people and assume an advisory role. The wide range of services (from pre-marriage issues to raising children) that midwives should be able to provide necessitate that they have a wide range of capabilities, and this need providing comprehensive training for midwives [26].

The second theme derived from the focus groups in our study was Manpower management which involved the following subthemes: Supplying skilled midwives, Midwifery management, and Support and incentive system. Supplying experienced midwives was one of the issues frequently mentioned by participants. In this respect, the head of the midwifery association pointed out that according to the law approved by the Ministry of Health, there must be 12 midwives for every 1000 live births in hospitals, but at the moment, only 8–9 midwives are available. It is worth mentioning that of the 8000 employed midwives, 2700 are temporarily employed (after graduation, each midwife can work in hospitals or health centers for 2 years and then can continue working if she is accepted in the employment exam). Therefore, after two years, most midwives become unemployed [27]. This is despite the fact that the need for midwives and midwifery care and services is on the rise, and more midwives should be employed.

The third subtheme of the “Manpower management” was related to the support and incentive system for midwives. The salary of midwives was among the most important issues raised by the participants. They believed that without good salary payments for the midwifery services, they would not have sufficient motivation and inclination to provide high-quality services. In recent years, Iranian officials have considered certain incentives for normal vaginal deliveries. However, the amount of this incentive for midwives is much lower than that for the obstetricians when they handle a normal vaginal delivery in a similar fashion [28, 29]. A performance-based payment was the topic that most of the participants mentioned in this regard, and they believed that most midwives are not satisfied with the salary they receive in hospitals and health care centers.

In this regard, the head of midwifery scientific association stated that more than 450,000 normal deliveries were attended by midwives last year, but only 15% of the delivery incentives were paid to them. She also added, “the delivery incentives need to be fairly distributed, yet contrary to our expectations, not only has no raise been considered in the salary payments of midwives, but their predefined rights have also been ignored”. Almost all fees are paid to on-call obstetricians, not to the midwives who care for mothers from the first stage of labor to the end. Since workplace discrimination and injustice disappoints the staff and reduces their motivation, accurate and fair systems should be designed and implemented.

The third theme in the present study was Rules, regulations, and standards of midwifery services which included three subthemes, namely regulations on professional competence of midwives, audit and supervision, and equipment and facilities. Midwives should be qualified to meet the changing health needs of women, mothers and children. They need to be equipped with cutting-edge and creative approaches in order to remain competitive and efficient to meet the needs of the modern societies [30]. Our results also indicated that midwives should adapt their knowledge and practice according to international standards.

Lack of sufficient equipment and facilities in hospitals and maternity wards was one of the frequent complaints of the participants. The results of previous Iranian studies in this field also indicate that physical space and facilities of the maternity wards in hospitals are not very satisfying, and all women, with high or low risk pregnancies, receive the same medically oriented care model offered by obstetricians, exposing them to unnecessary intervention and bringing about the possibility of cesarean section [31, 32]. Sanitation and provision of an appropriate environment for normal delivery can be influential in the satisfaction of mothers and consequently in the delivery pattern [33]. A qualitative study by Shirzad et al. showed that physical condition of health systems, differences between private and public hospitals, and lack of access to pain relief during vaginal delivery are amongst the most important factors that may influence women’s decision about mode of delivery [34].

The fourth theme extracted in the present study was Policy making and which included the following subthemes: Organization, Compliance with general policies, and Interdisciplinary activities. Participants mentioned that the position of the midwife in the referral system is not clear and should be clearly defined. The family doctor plan has been implemented in several provinces of Iran for 15 years. According to this plan, there should be a doctor and a midwife in the health systems, and each patient can be referred to a specialist with the approval of the family doctor. The results of studies on the family doctor plan indicate many weaknesses and problems in the implementation of this plan and its referral system [35]. Participants in this study emphasized the presence of midwives and their bold and predefined role in the family doctor team. In this regard, some of the participants were of the opinion that midwives should have a pronounced and executive role from the pre-marriage stages until the raising of the children, and this role continues even to the middle age.

In the study of Lotfi et al. aimed at presenting strategies to reduce the cesarean rate in Iran, emphasis was put on revisiting the roles of midwives and specifying new fitting roles for them. Moreover, there is evidence that a pregnant woman should be visited by a midwife and referred by her if necessary [35].

In the present study, one of the issues raised by participants was the obstetricians’ performing cesarean sections without any obvious indication. They believed that according to some gynecologists’ opinion, normal delivery is very time consuming while cesarean section takes them only one hour to perform. The strategies the participants proposed in this respect included imposing strict and efficient punishment and establishing a monitoring system to control the practice of the gynecologists in hospitals. Faraji et al. [36] found that in 70% of cases, gynecologists played the main role in the high cesarean section rate in Iran. A study by Menacker et al. [37] attributed the increase in the rate of cesarean section with no indication to the tendencies and the judgment of doctors. Perhaps the high fees of cesarean surgery compared with the normal delivery subconsciously motivate doctors to perform more cesarean operations. In this regard, the plan to implement hospital incentives in Iran can have a great impact. Moreover, the development of self-control and monitoring tools and methods in this regard could be useful.

As far as interdisciplinary teamwork is concerned, the improvement of the collaboration between midwives and gynecologists was a topic repeatedly mentioned by the participants. In the study of Lotfi et al. interdisciplinary collaboration is reported to have a role in the reduction of cesarean rate [35]. The effective collaboration among groups of professionals has increasingly been the focus of attention as a fundamental element in safety and good quality of health care. This is especially significant in the context of maternal care, where women have the experience of delivery [38, 39].

According to the results of some recent studies, the most important obstacles of teamwork are as follows: lack of specific task limits for each person, weak management, inconsistent communication, the presence of a hierarchical relationship in wards, insufficient skill and knowledge, and inappropriate division of responsibilities [40, 41].

The main limitation of the current study is that high-ranked authorities and policymakers of the Ministry of Health and Medical Education failed to participate in it. Second, in this study, only five out of 121 participants were gynecologists, and although we reached saturation with the current numbers of participants, this might have caused a bias.

## Conclusion

The results of this study showed that in order to improve midwifery care, emphasis should be put on the quality and quantity of midwifery education, training individuals and informing them, and increasing midwifery human resources through employment. Furthermore, insurance support, encouragement, supporting and motivating midwives, enhancing and improving the facilities and providing hospitals and maternity wards with cutting-edge equipment, promoting and reinforcing the position of midwives in the family doctor plan, and using a referral system have been mentioned by the participants as viable strategies for improving midwifery care. Finally, establishing an efficient and powerful monitoring system to control the practice of gynecologists and midwives, increasing the collaborative practice of midwives and gynecologists, and promoting teamwork with respect to midwifery care are other strategies to improve the midwifery service in Iran.

## Supplementary Information

**Additional file 1.** Focus group discussion guide.

## Data Availability

Data of this study will be available upon the request from the corresponding author.

## Abbreviations

WHO: World Health Organization
IPA: Involvement and Participation Association
IHE: Institution of Health Equality
NHS: National Health Service
