# Supplementary material for: Strategies to improve the quality of midwifery care and developing midwife-centered care in Iran: analyzing the attitudes of midwifery experts
Source: BMC Pregnancy Childbirth. 2022 Jan 16;22:40. doi: 10.1186/s12884-022-04379-7 (PMC8762813; doi:10.1186/s12884-022-04379-7)
Supplement: Supplementary file 1 — Additional file 1. Focus group discussion guide. [file 12884_2022_4379_MOESM1_ESM.docx]

A guide to focus group discussion was developed to conduct this study as follows:

**Group details:**

1. Voluntary participation in the group
2. Homogeneity of the group
3. Number of group members

**Identify the characteristics and role of key people**

1. Features and role of facilitator
2. Characteristics and role of the group secretory
3. The do’s and don’ts of a facilitator or secretory

Time and place of meetings and characteristics of the place (same interview location for all groups except for gynecologists and midwifery board)

Meeting during office hours and meetings are limited to a maximum of 90 minutes with a reception in the middle of the meeting

**Holding meetings**

1. Provide an introduction for all groups
2. Explain the purpose of the study
3. Explain about the agreement of people to participate in the meeting.
4. Brief explanation of the role of individuals and how to manage the discussion and rules related to group discussion
5. Presenting questions
6. Holding a discussion
